# Supplementary figures and images for: GSDMD suppresses keratinocyte differentiation by inhibiting FLG expression and attenuating KCTD6-mediated HDAC1 degradation in atopic dermatitis
Source: PeerJ. 2024 Jan 16;12:e16768. doi: 10.7717/peerj.16768 (PMC10798152; doi:10.7717/peerj.16768)

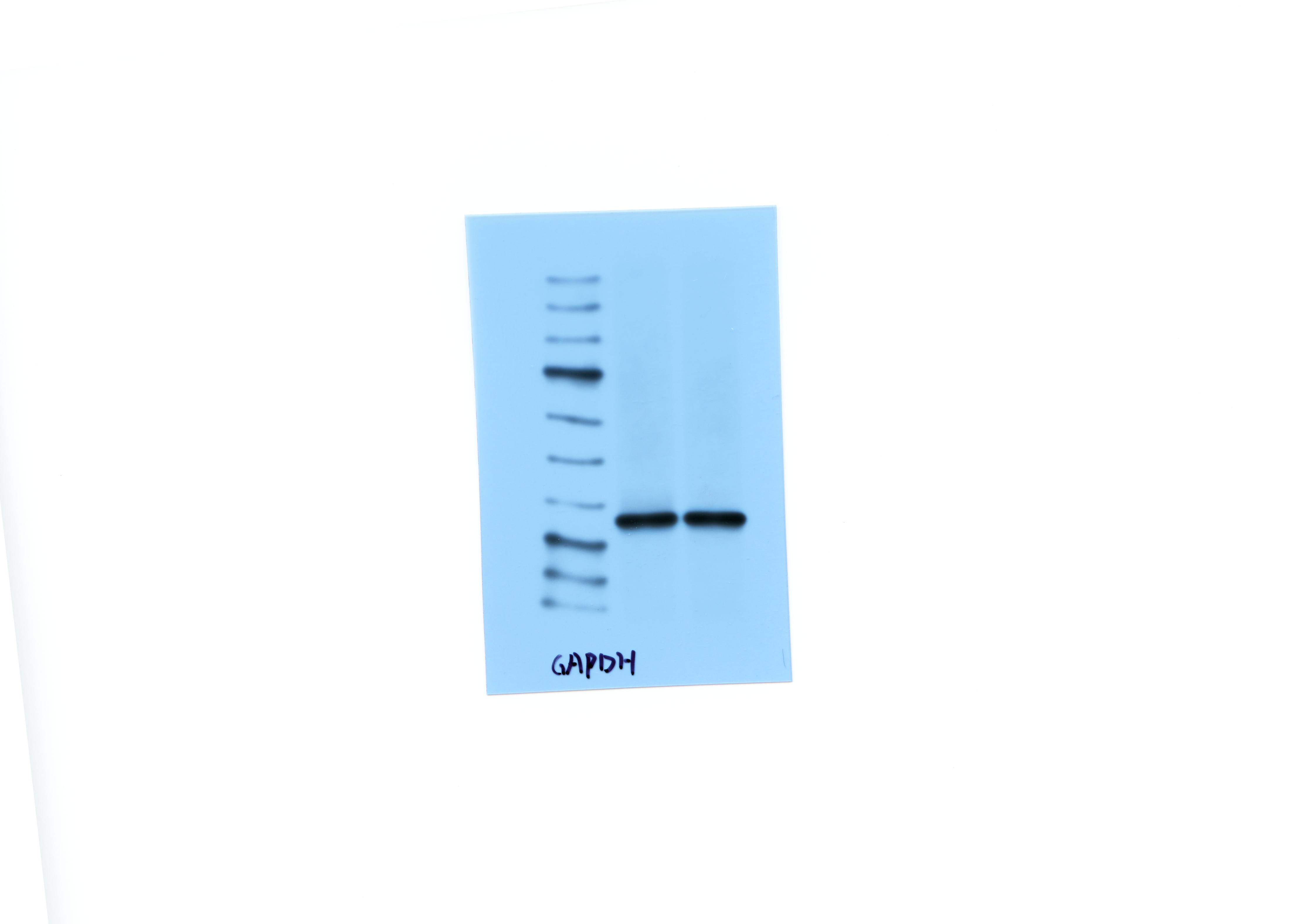

Supplement: Supplemental Information 2 [file peerj-12-16768-s002.zip › 3A-GAPDH (1).tif]

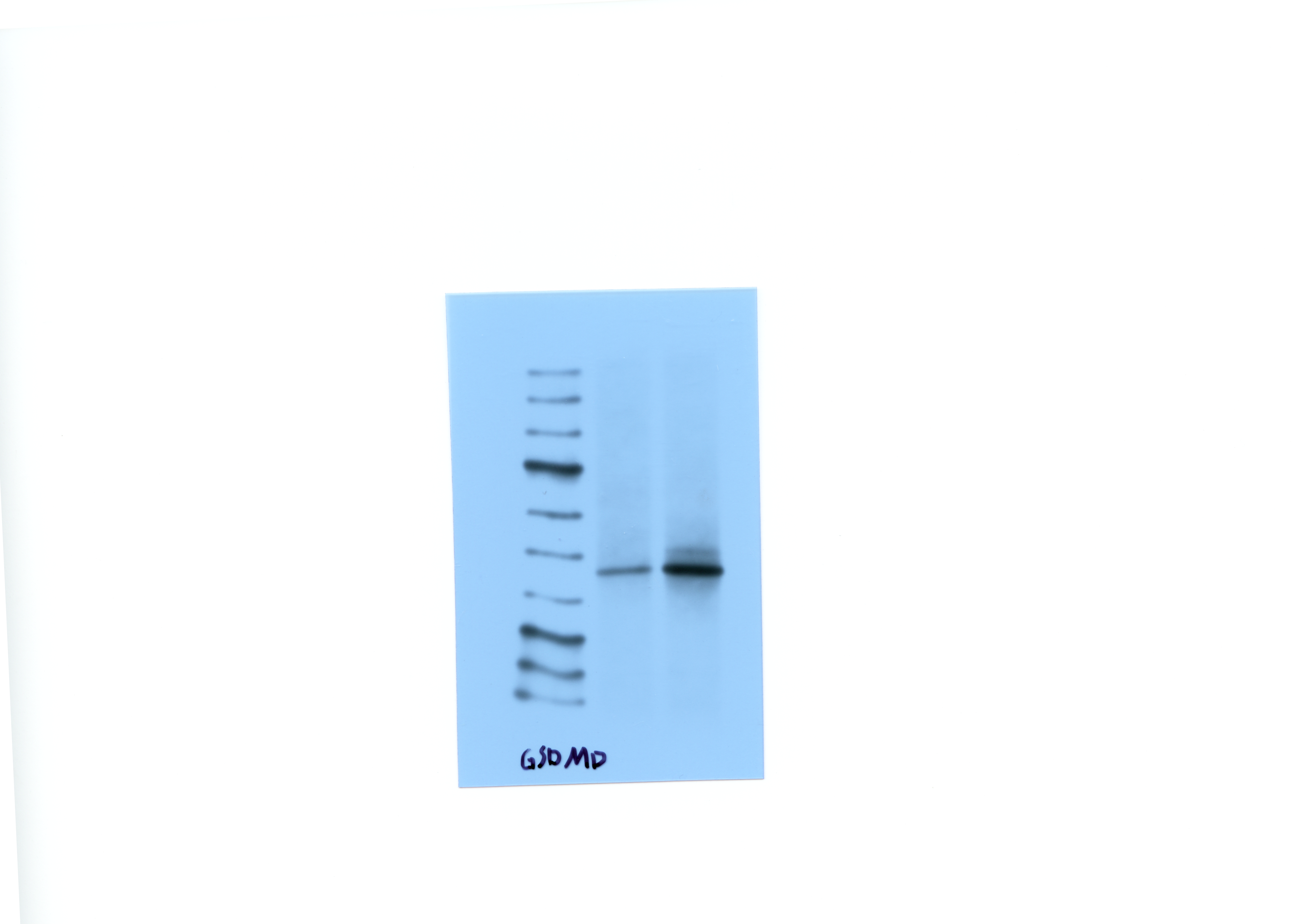

Supplement: Supplemental Information 2 [file peerj-12-16768-s002.zip › 3A-GSDMD (1).tif]

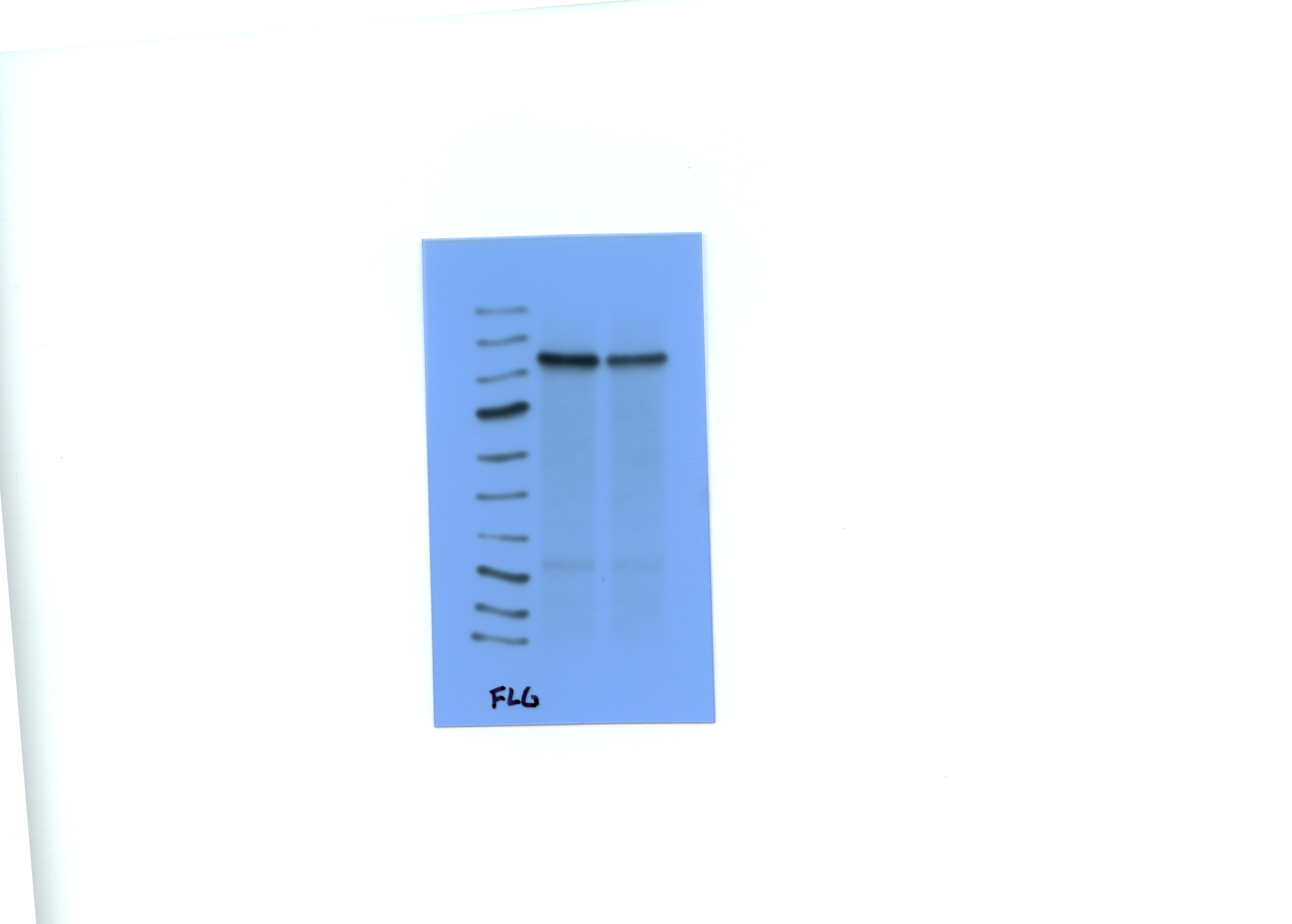

Supplement: Supplemental Information 3 [file peerj-12-16768-s003.zip › 3B-FLG (1).tif]

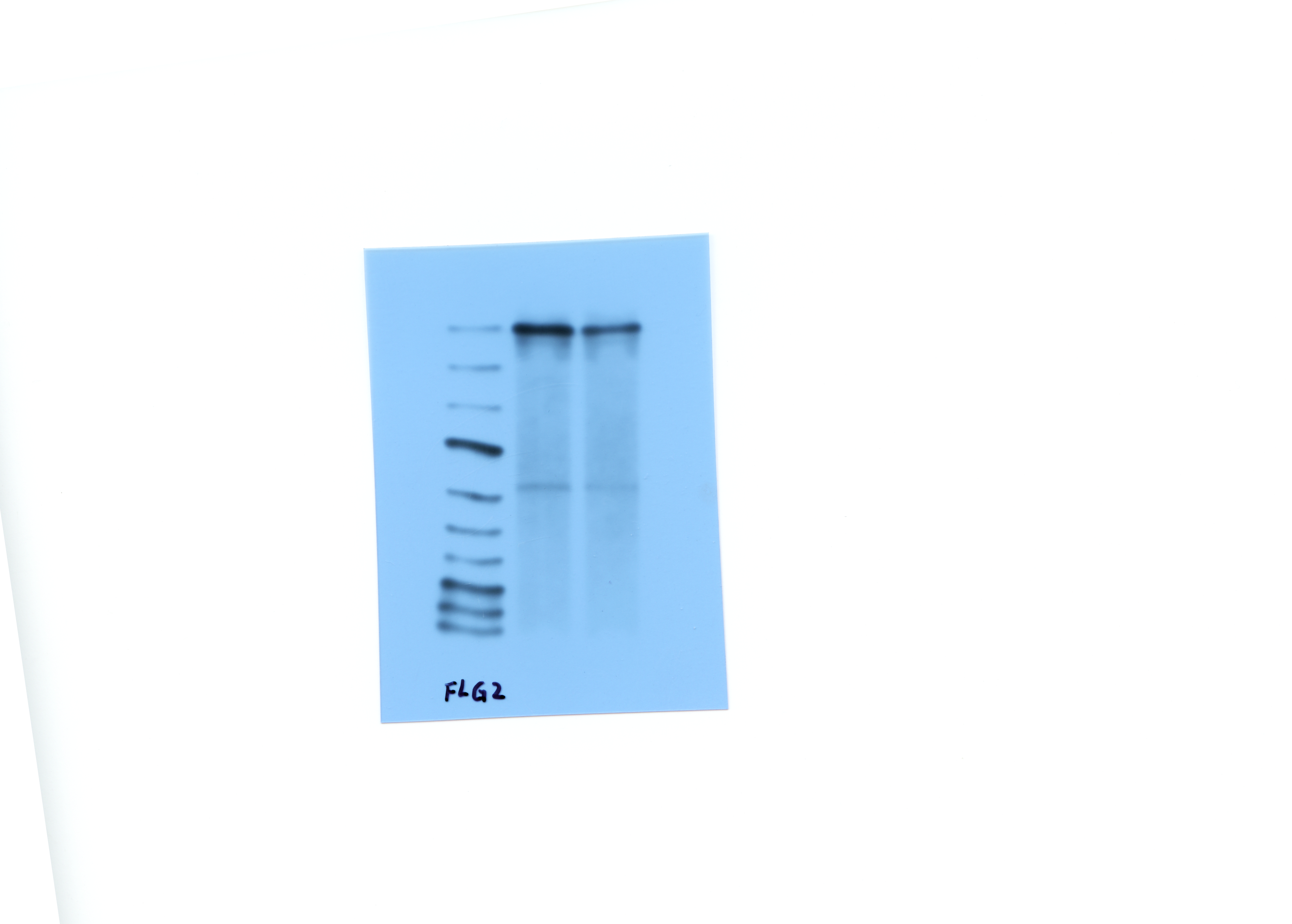

Supplement: Supplemental Information 3 [file peerj-12-16768-s003.zip › 3B-FLG2.tif]

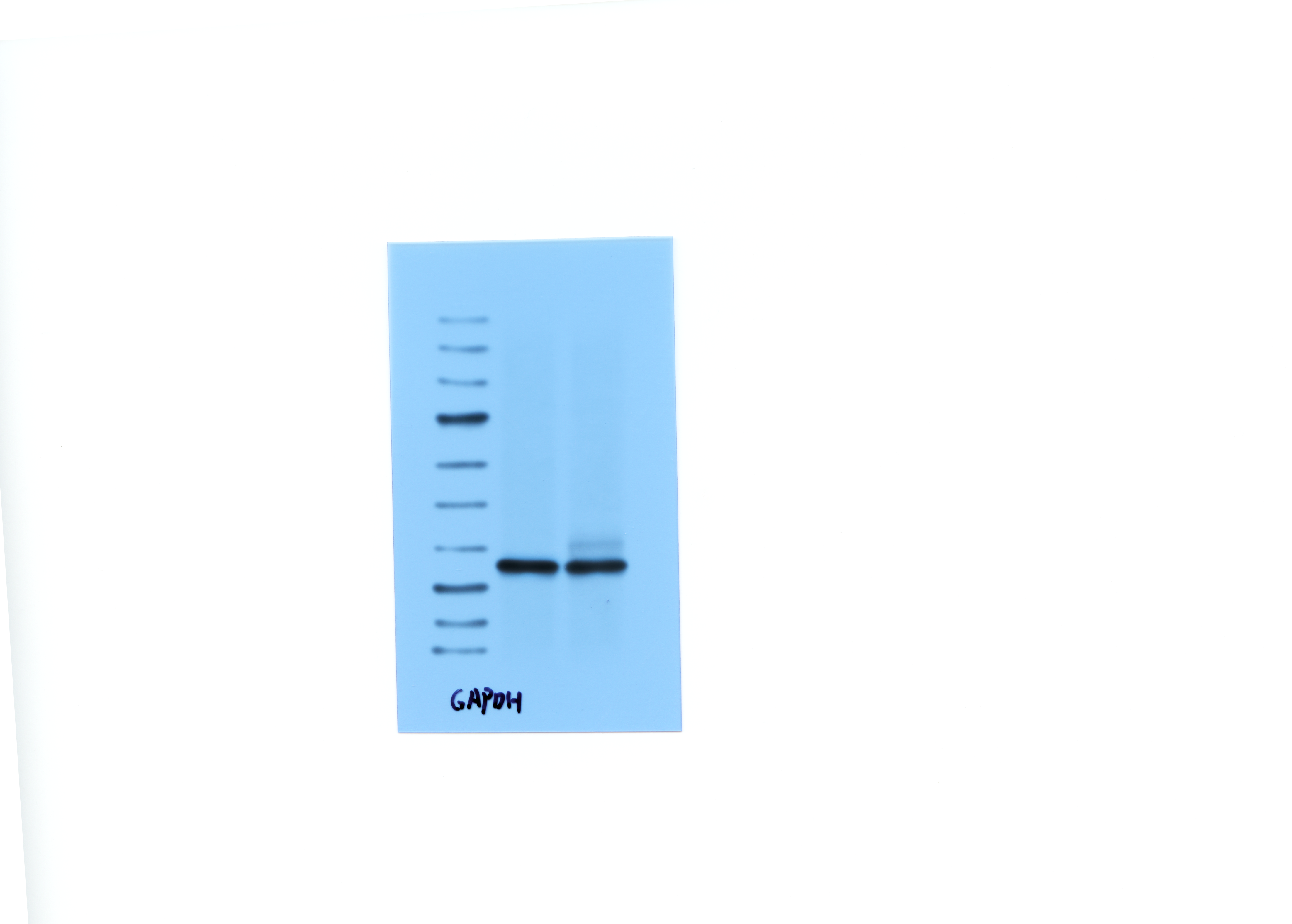

Supplement: Supplemental Information 3 [file peerj-12-16768-s003.zip › 3B-GAPDH (1).tif]

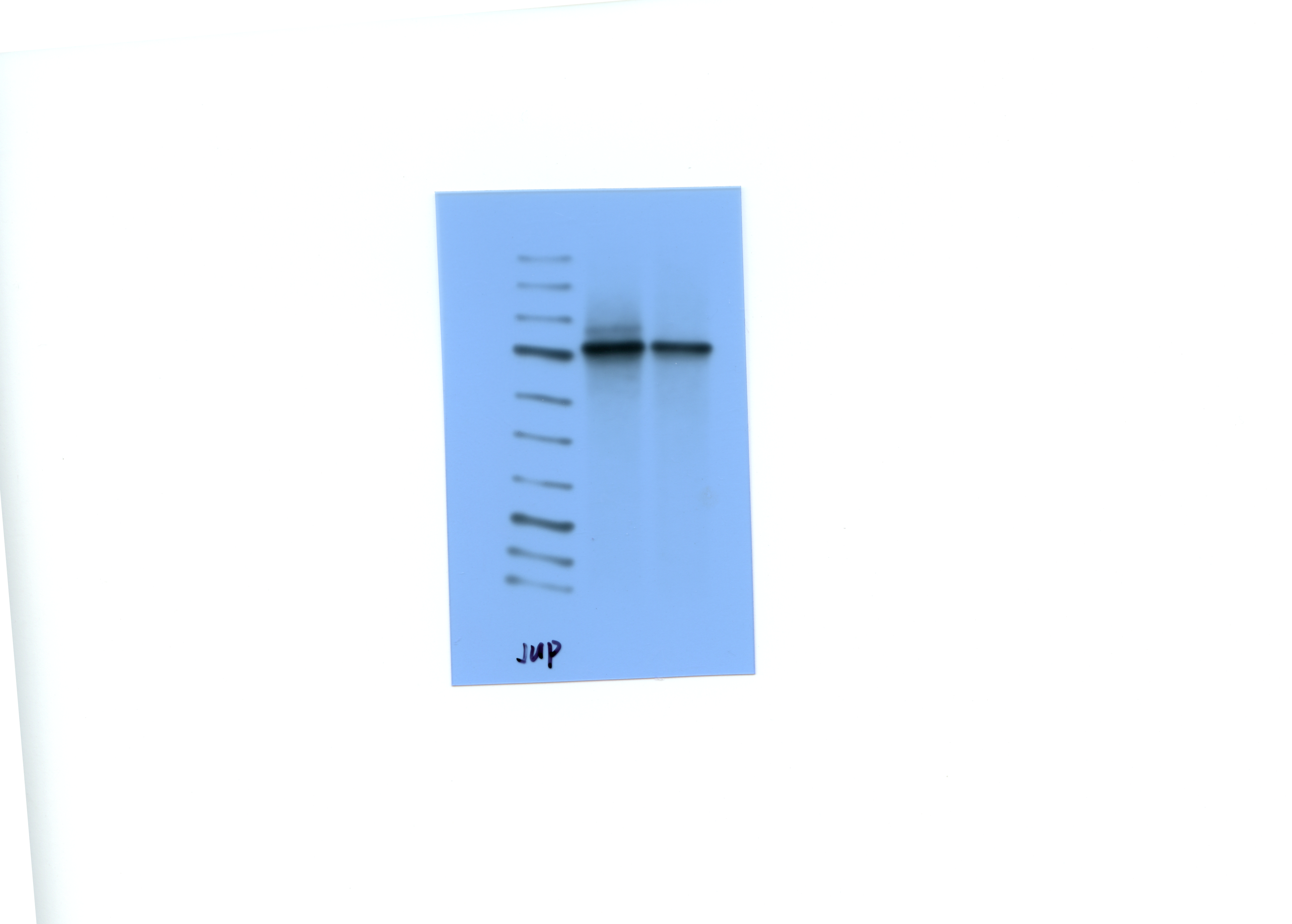

Supplement: Supplemental Information 3 [file peerj-12-16768-s003.zip › 3B-JUP.tif]

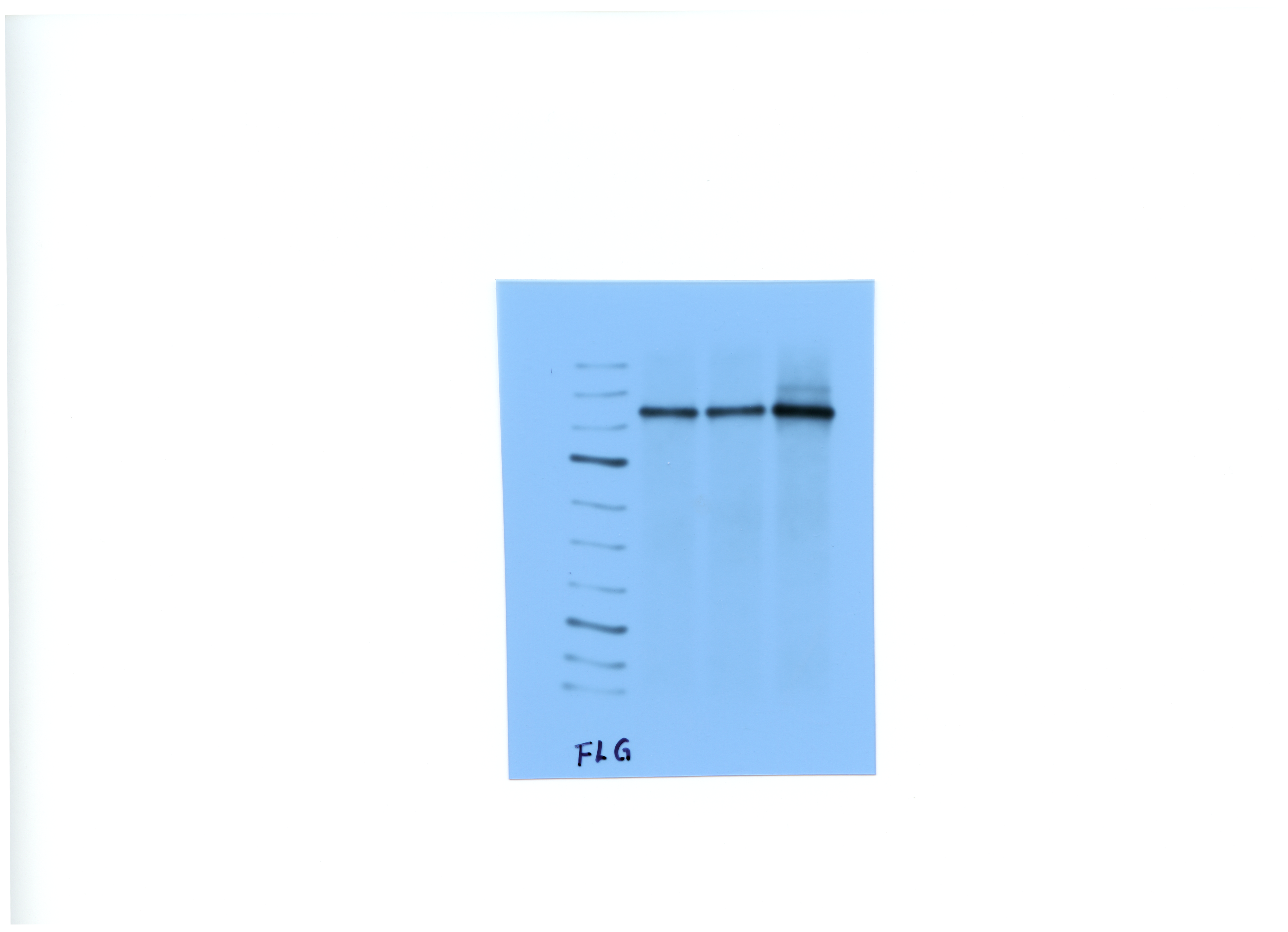

Supplement: Supplemental Information 4 [file peerj-12-16768-s004.zip › 4B-FLG.tif]

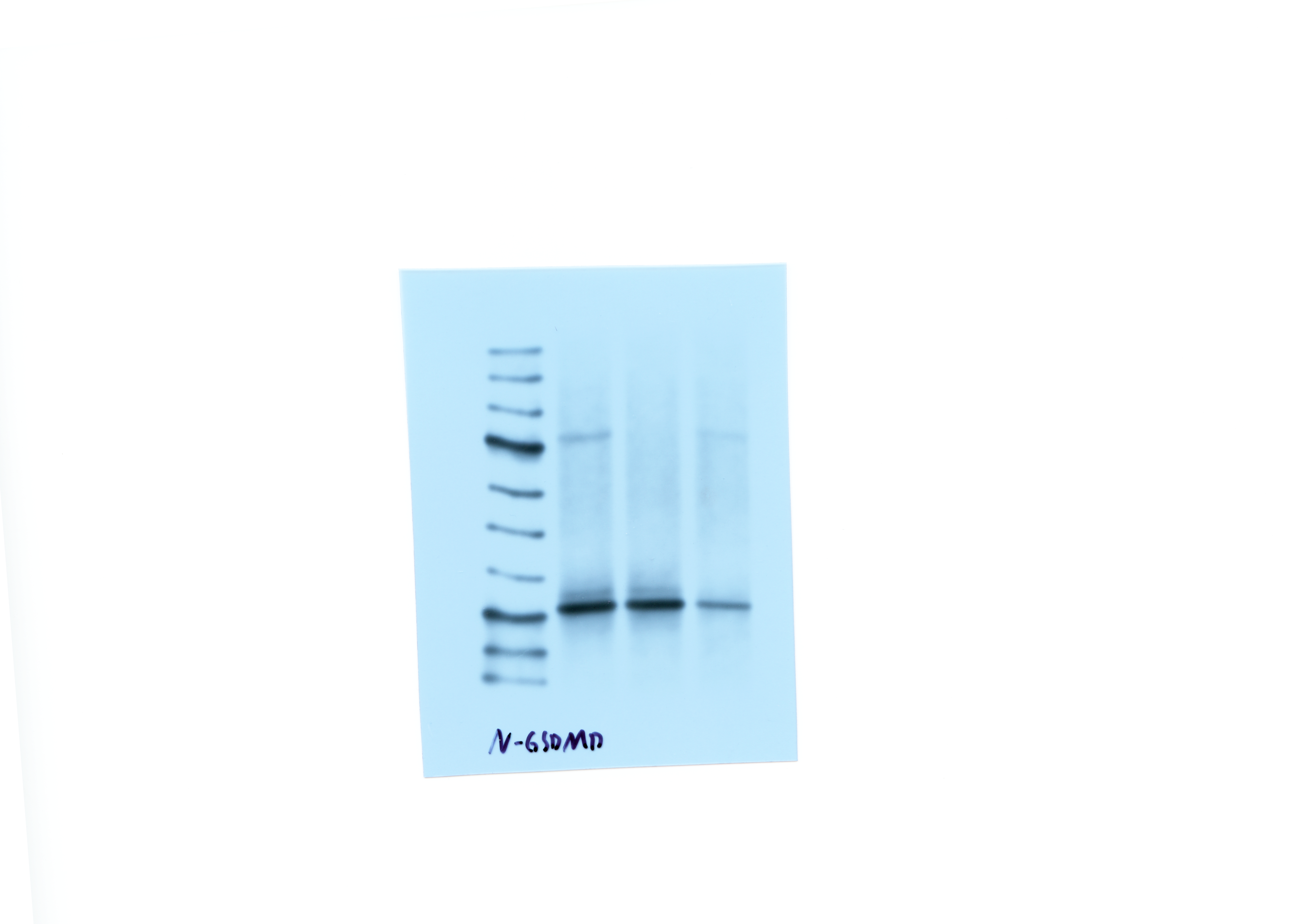

Supplement: Supplemental Information 4 [file peerj-12-16768-s004.zip › 4B-GSDMD.tif]

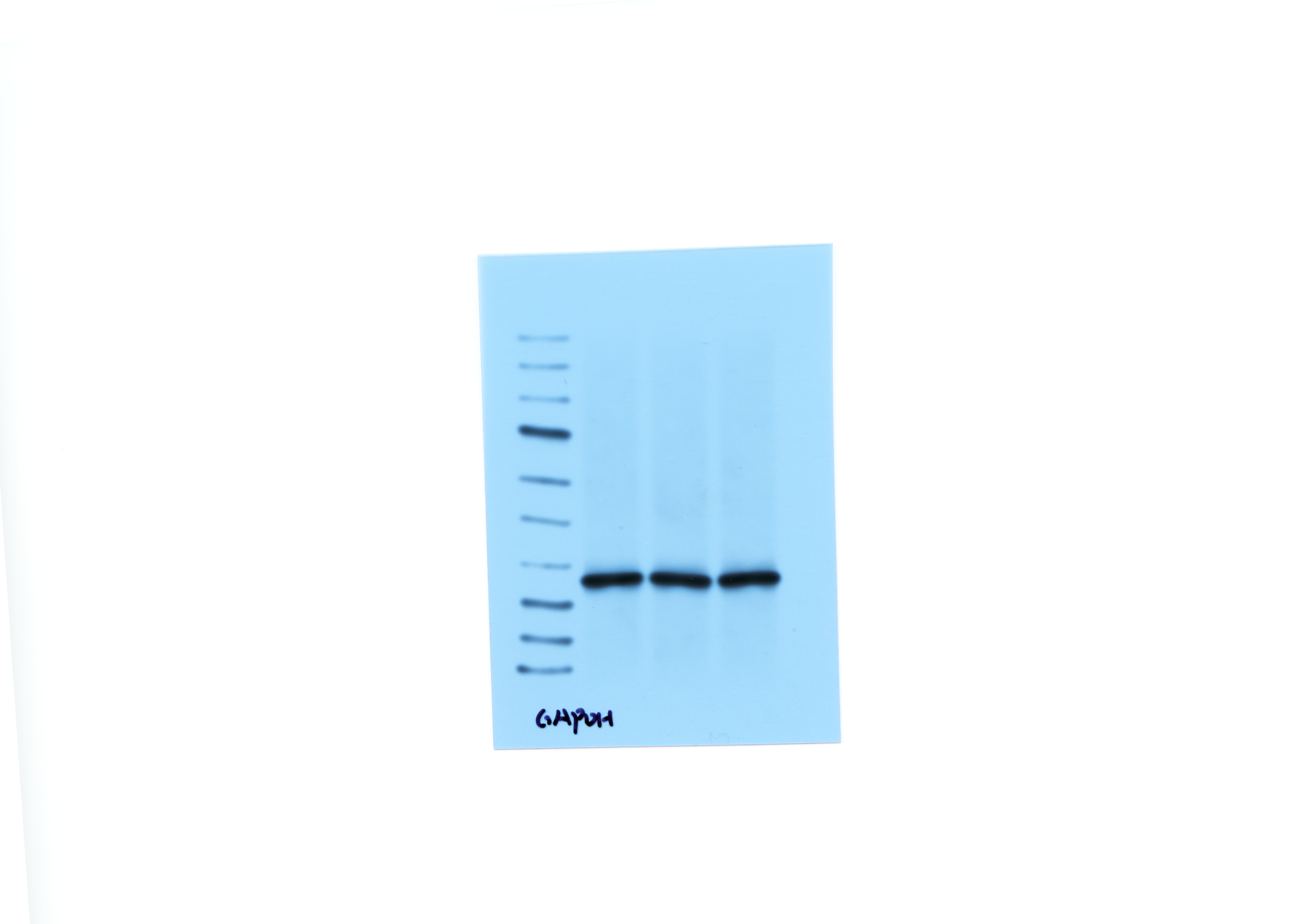

Supplement: Supplemental Information 5 [file peerj-12-16768-s005.zip › 4B-GAPDH.tif]

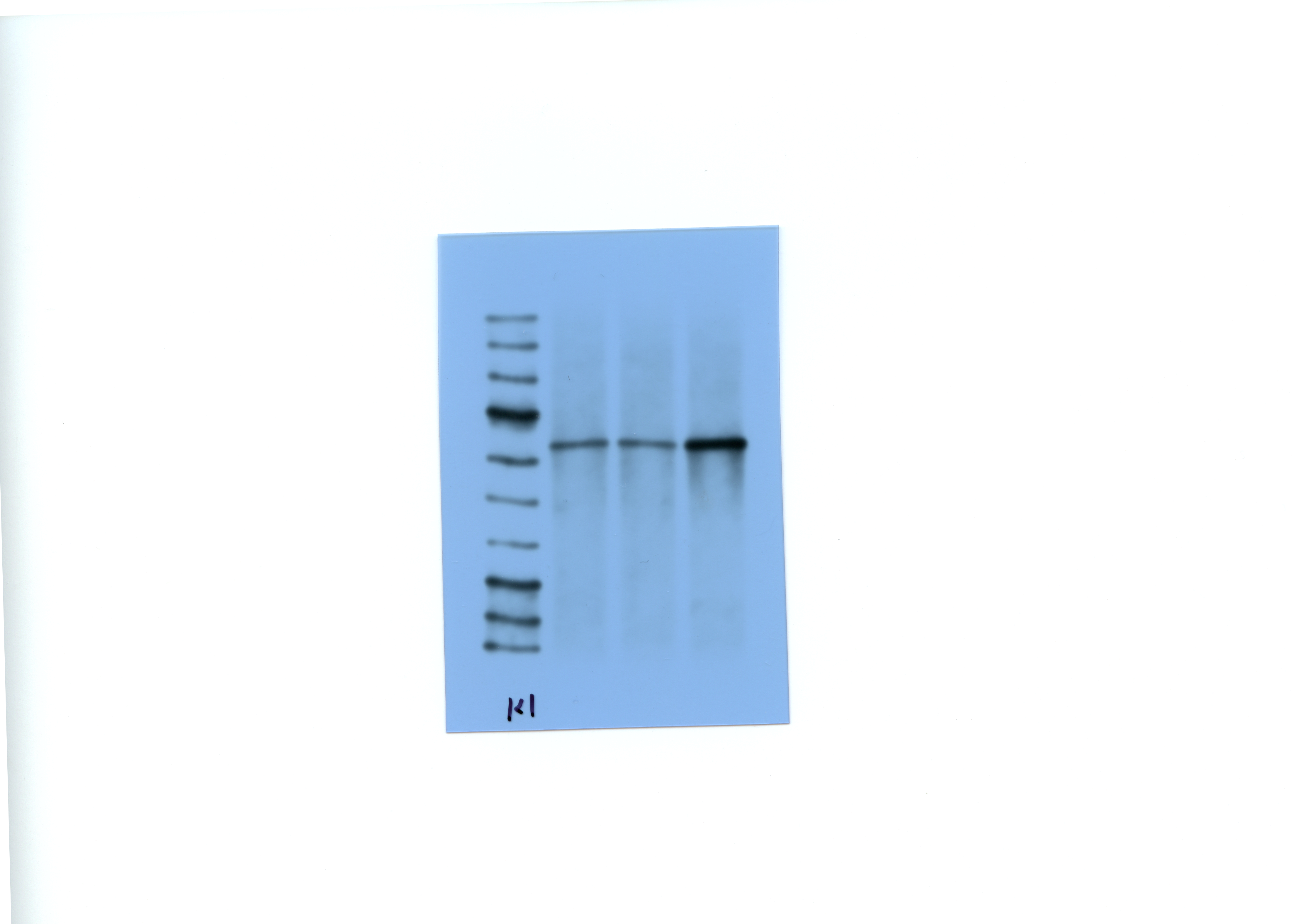

Supplement: Supplemental Information 5 [file peerj-12-16768-s005.zip › 4B-K1.tif]

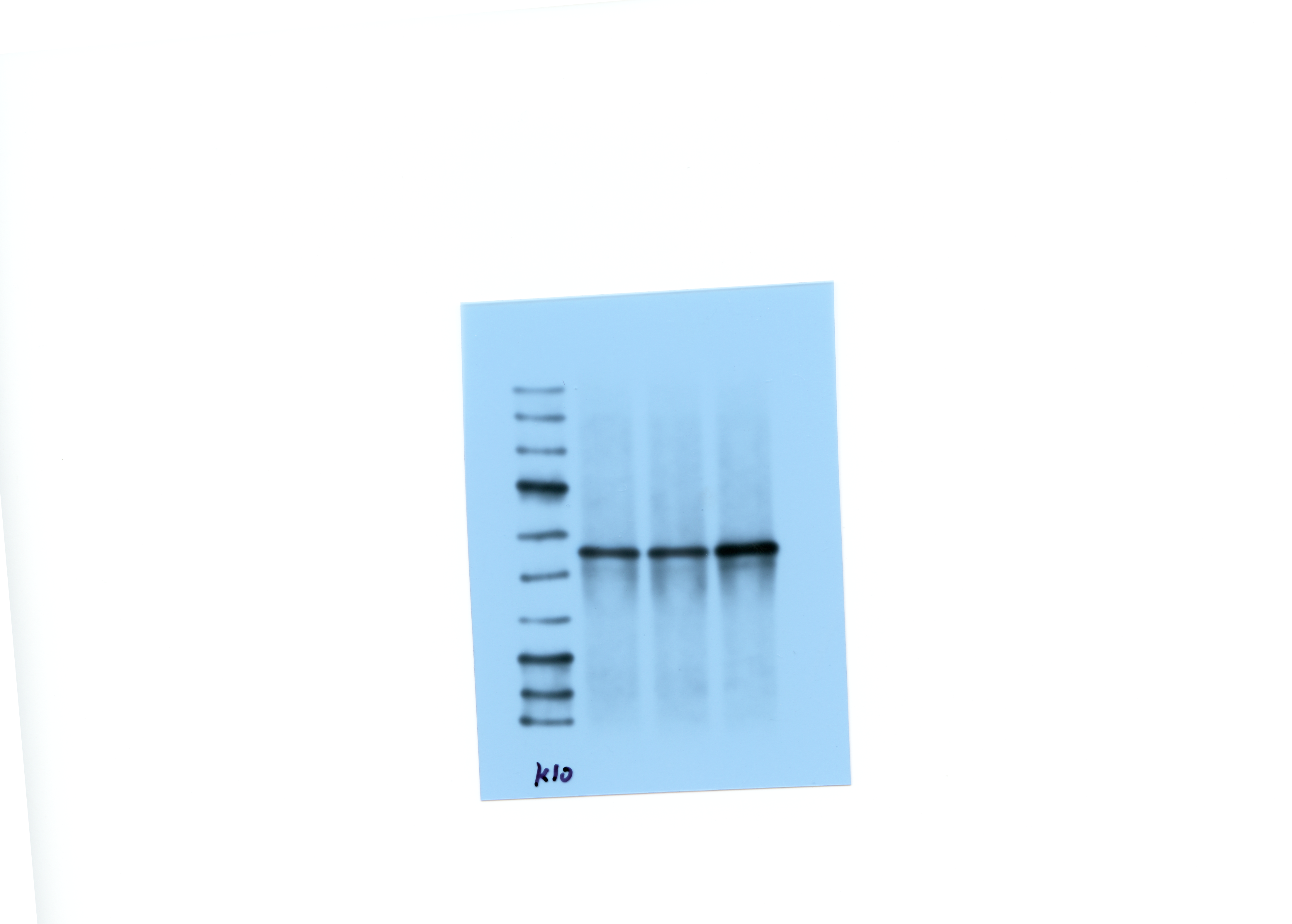

Supplement: Supplemental Information 5 [file peerj-12-16768-s005.zip › 4B-K10.tif]

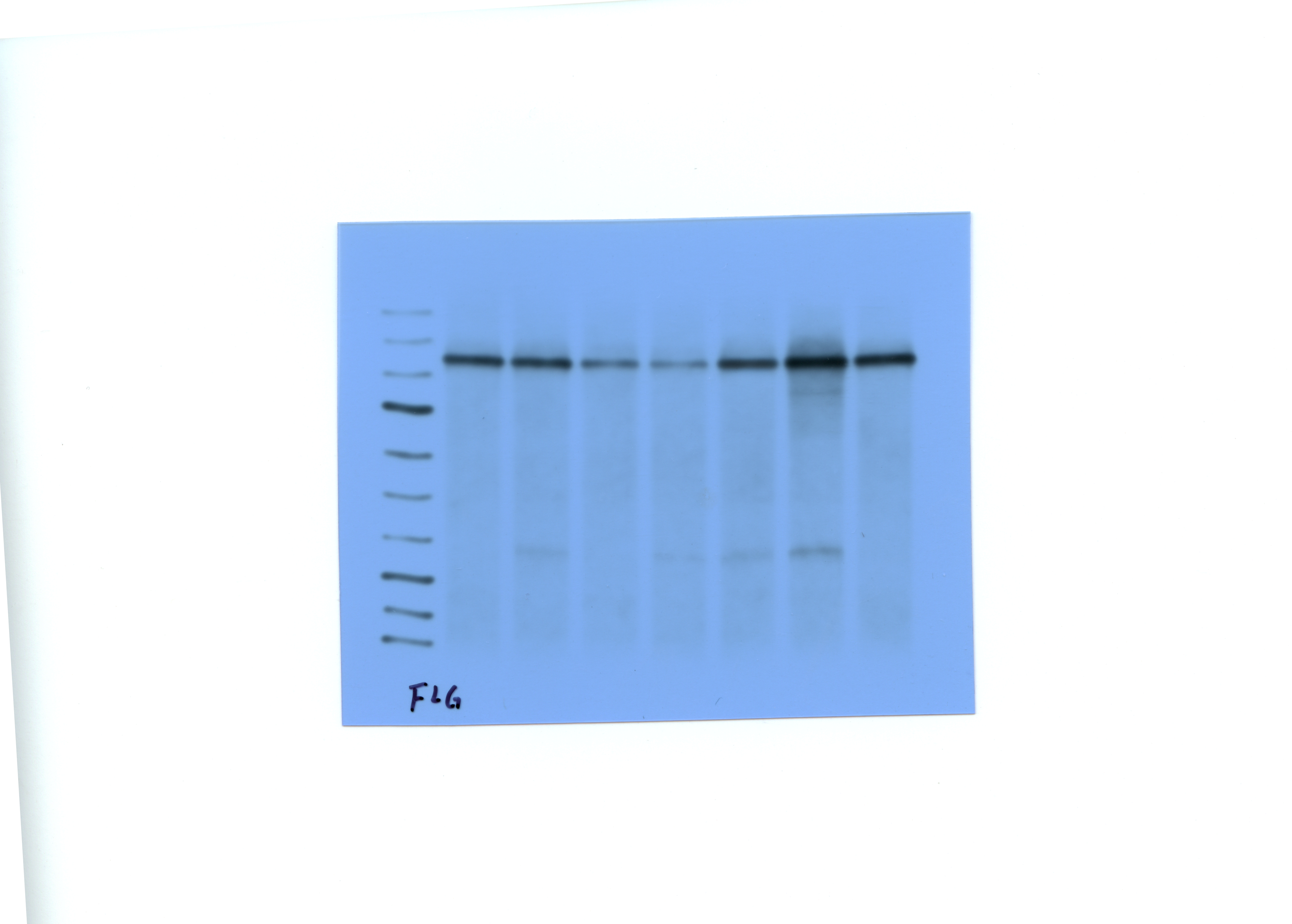

Supplement: Supplemental Information 6 [file peerj-12-16768-s006.zip › 5B-FLG.tif]

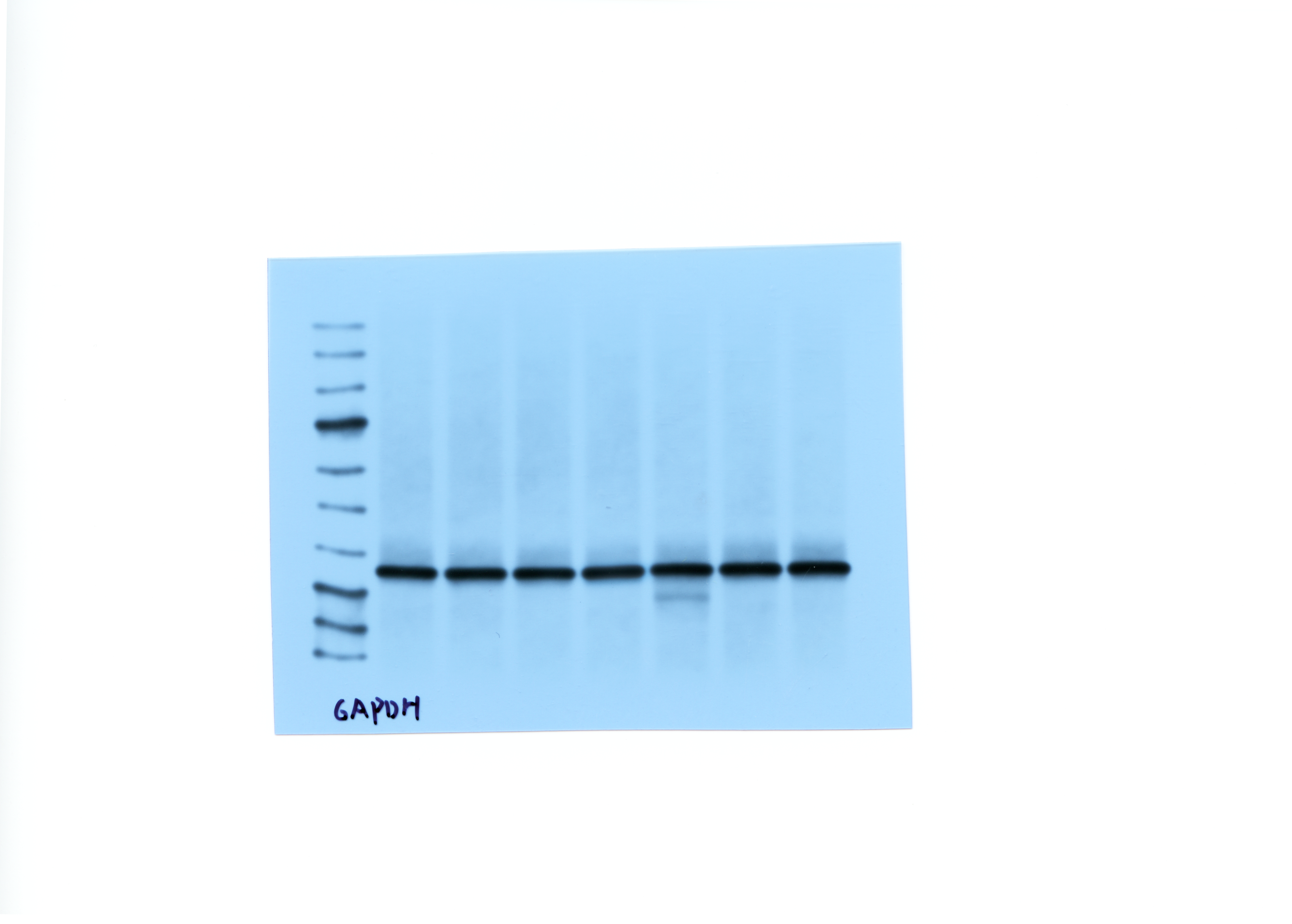

Supplement: Supplemental Information 6 [file peerj-12-16768-s006.zip › 5B-GAPDH.tif]

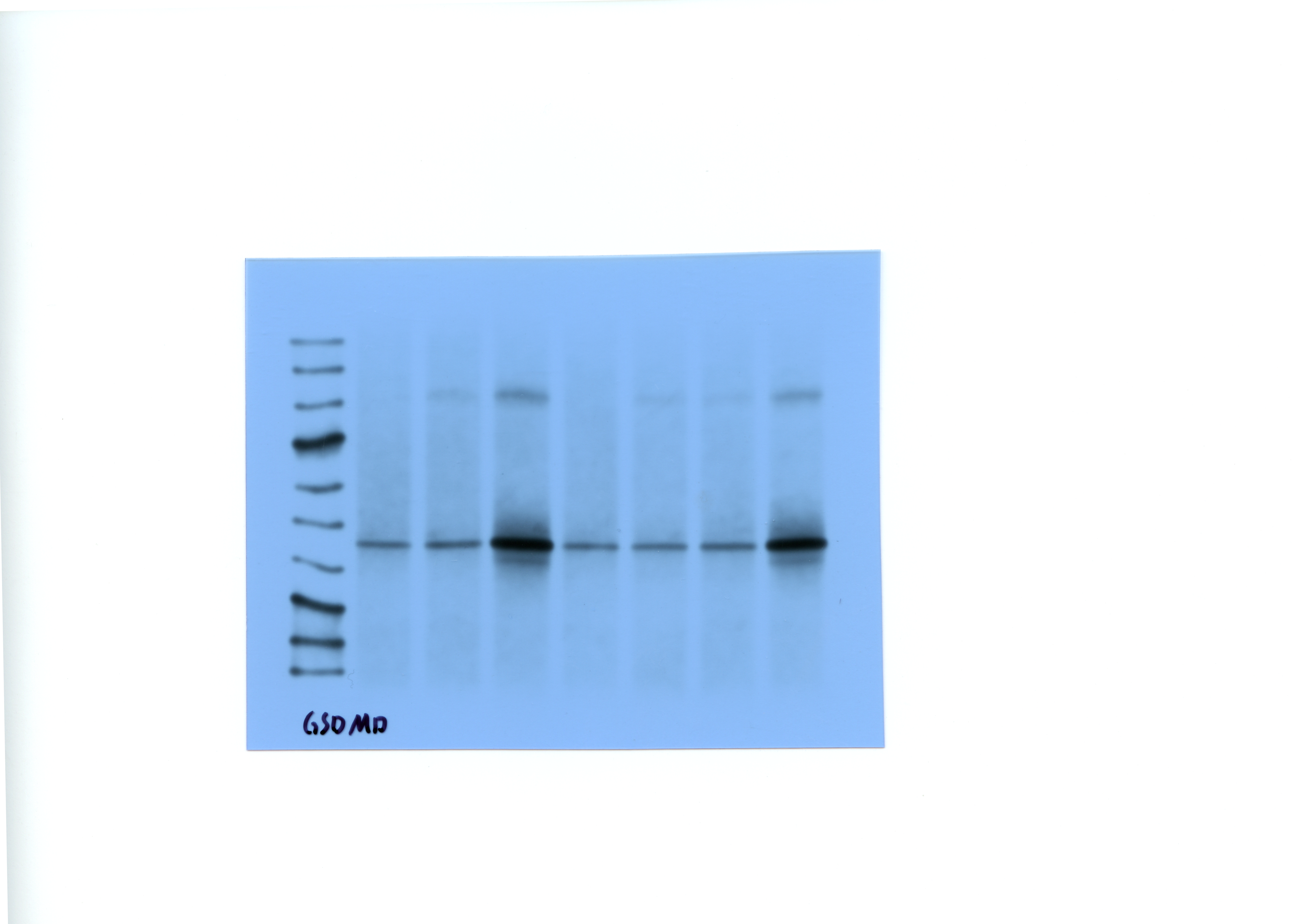

Supplement: Supplemental Information 7 [file peerj-12-16768-s007.zip › 5B-GSDMD.tif]

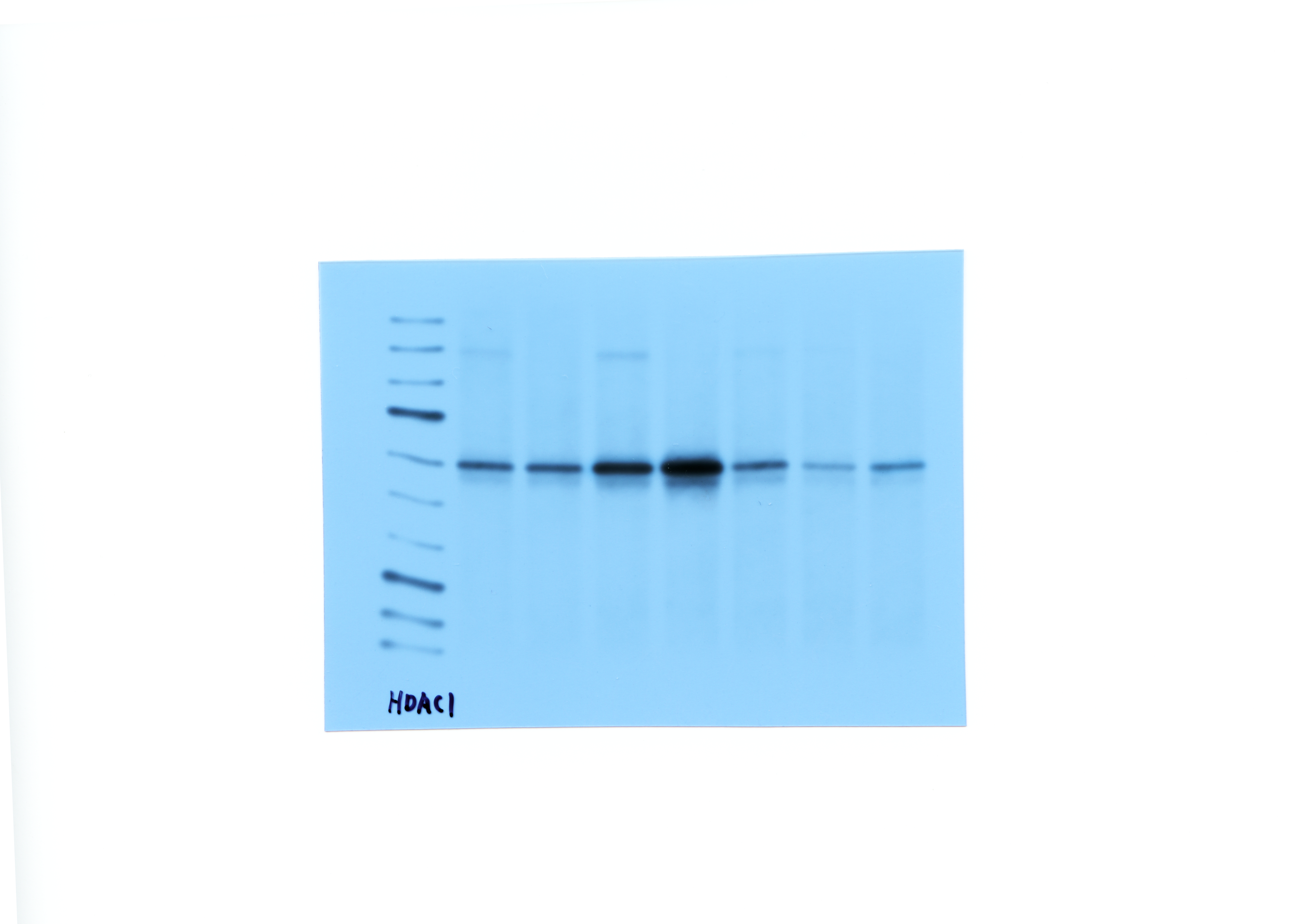

Supplement: Supplemental Information 7 [file peerj-12-16768-s007.zip › 5B-HDAC1.tif]

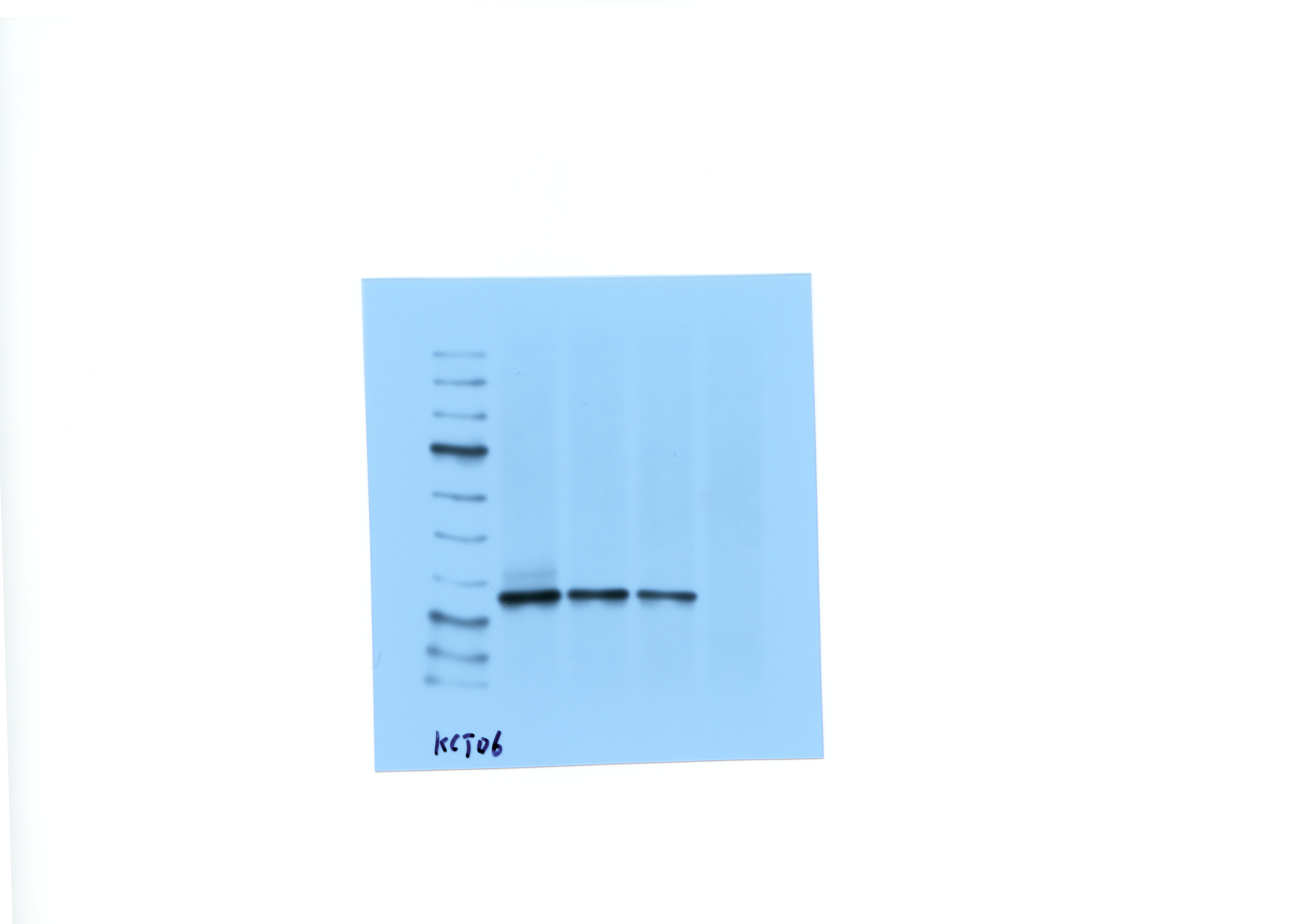

Supplement: Supplemental Information 8 [file peerj-12-16768-s008.zip › 6B-KCTD6.tif]

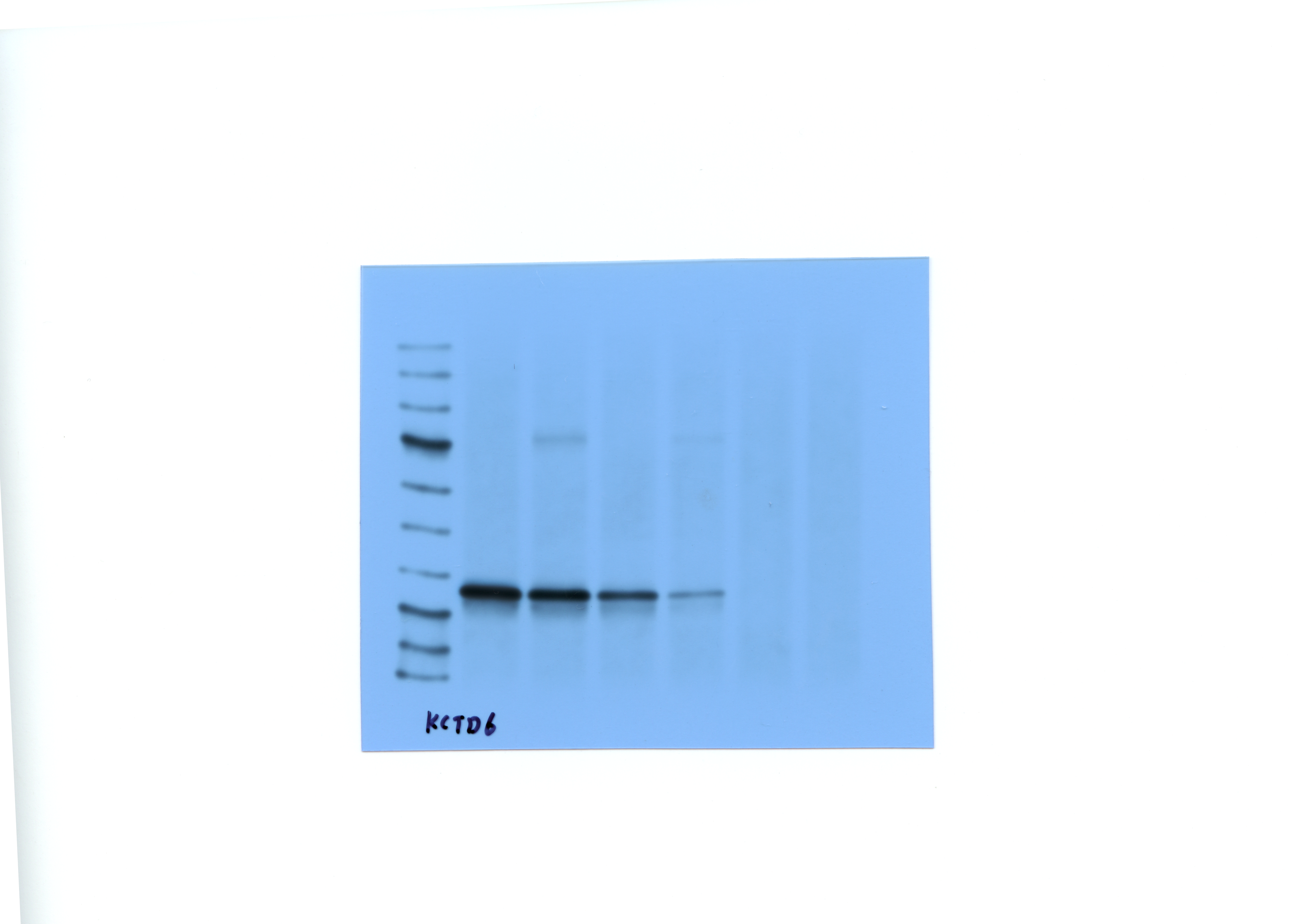

Supplement: Supplemental Information 8 [file peerj-12-16768-s008.zip › 6D-KCTD6.tif]

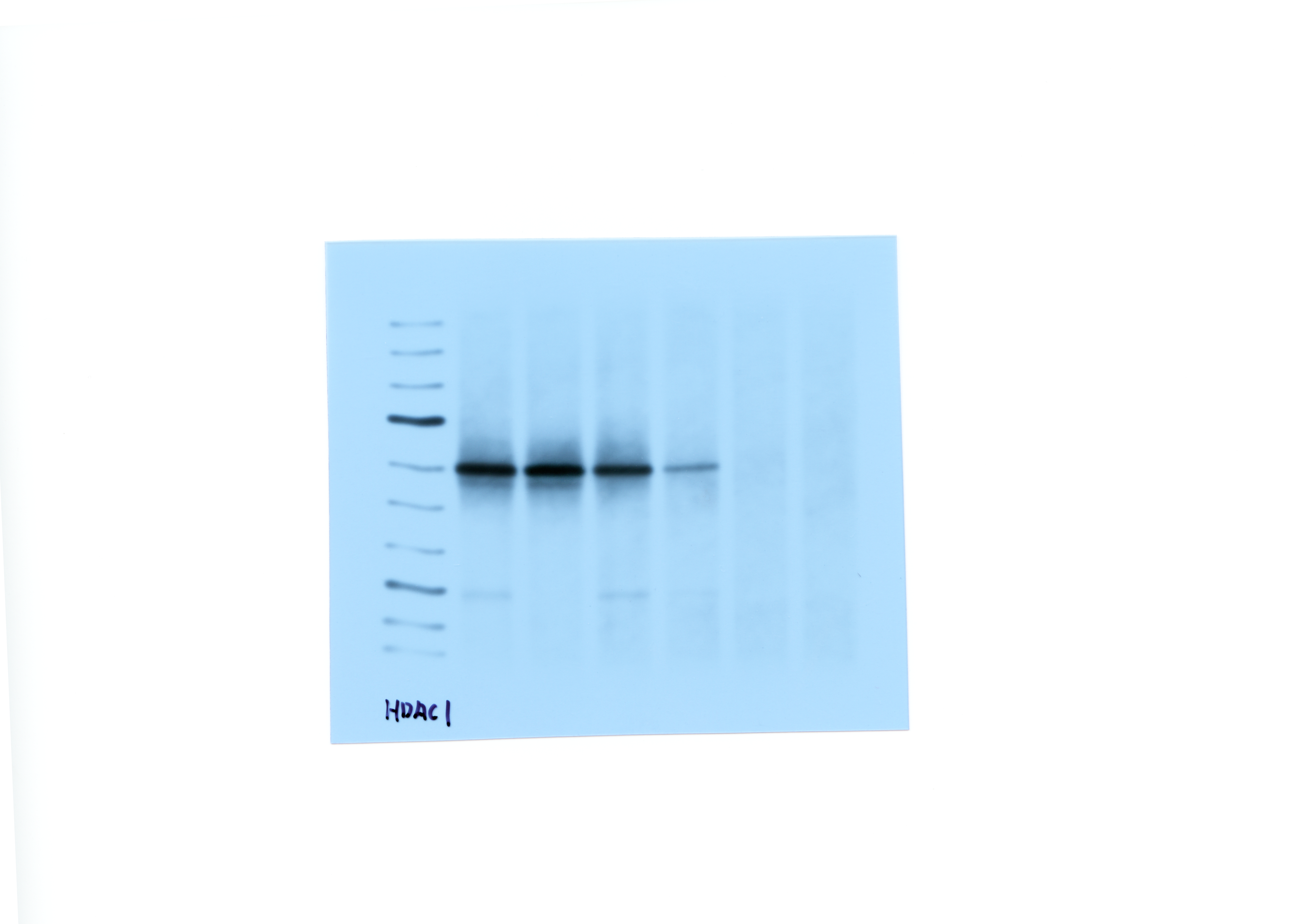

Supplement: Supplemental Information 8 [file peerj-12-16768-s008.zip › 6E-HDAC1.tif]

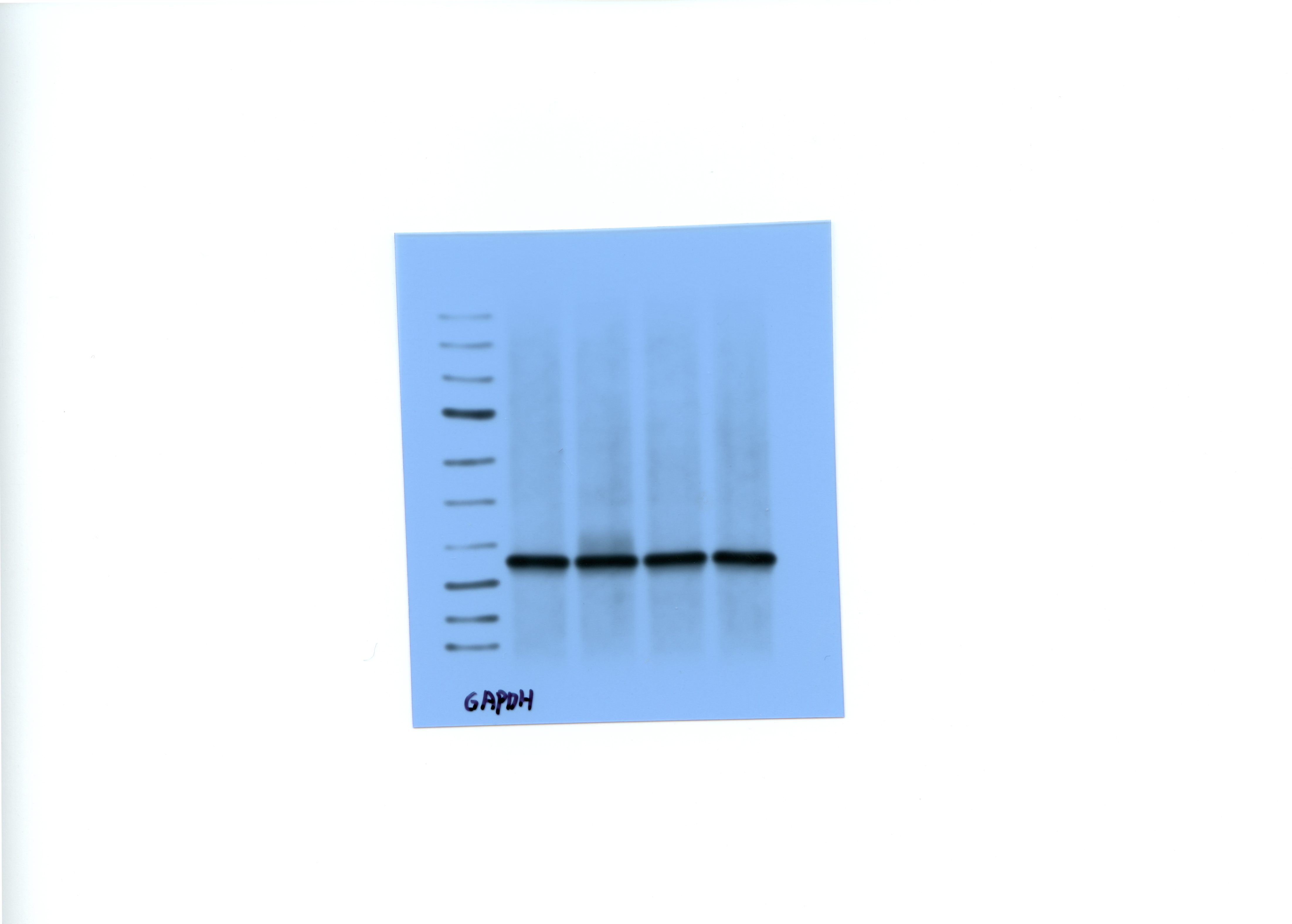

Supplement: Supplemental Information 9 [file peerj-12-16768-s009.zip › 6F-GAPDH.tif]

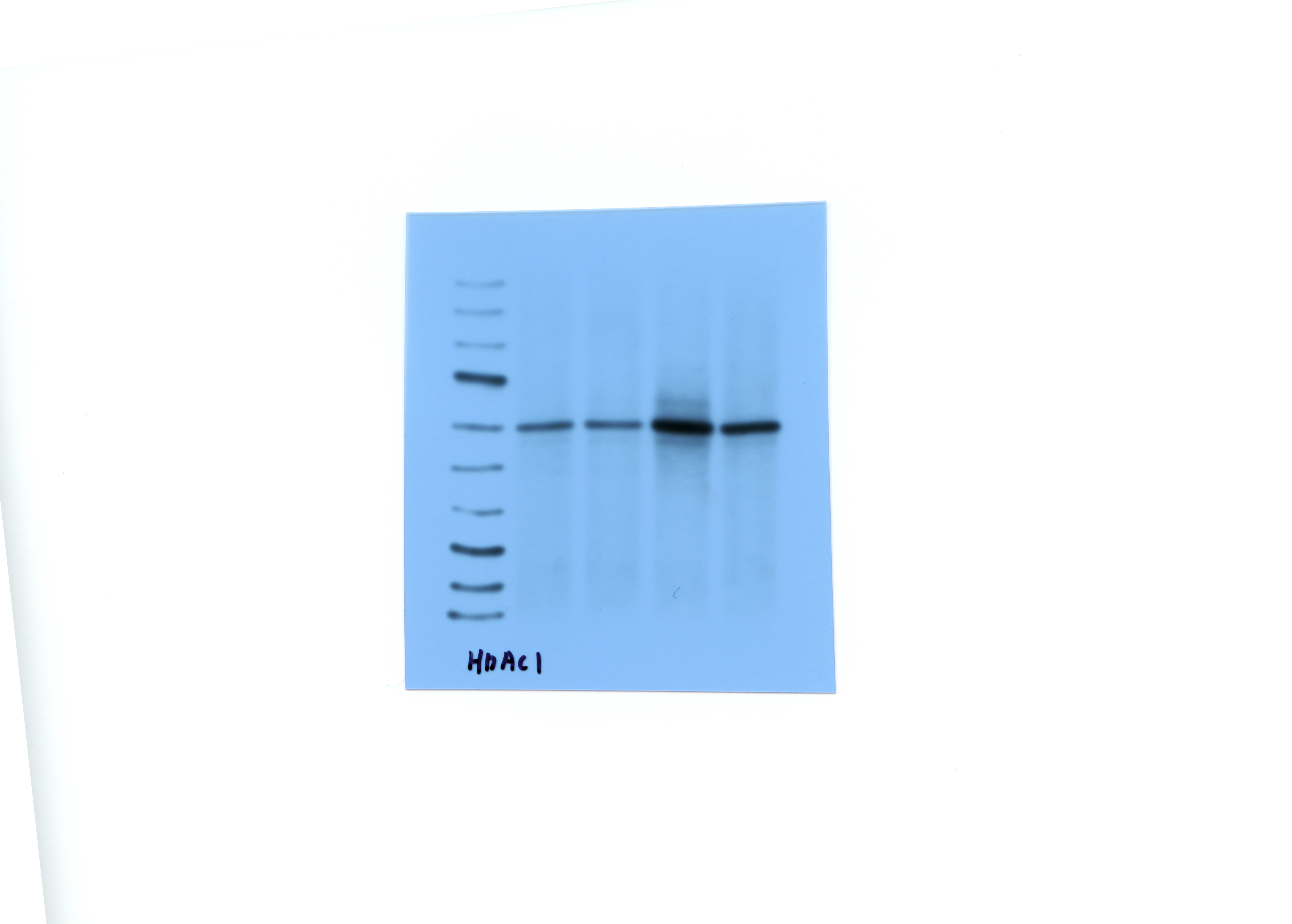

Supplement: Supplemental Information 9 [file peerj-12-16768-s009.zip › 6F-HDAC1.tif]

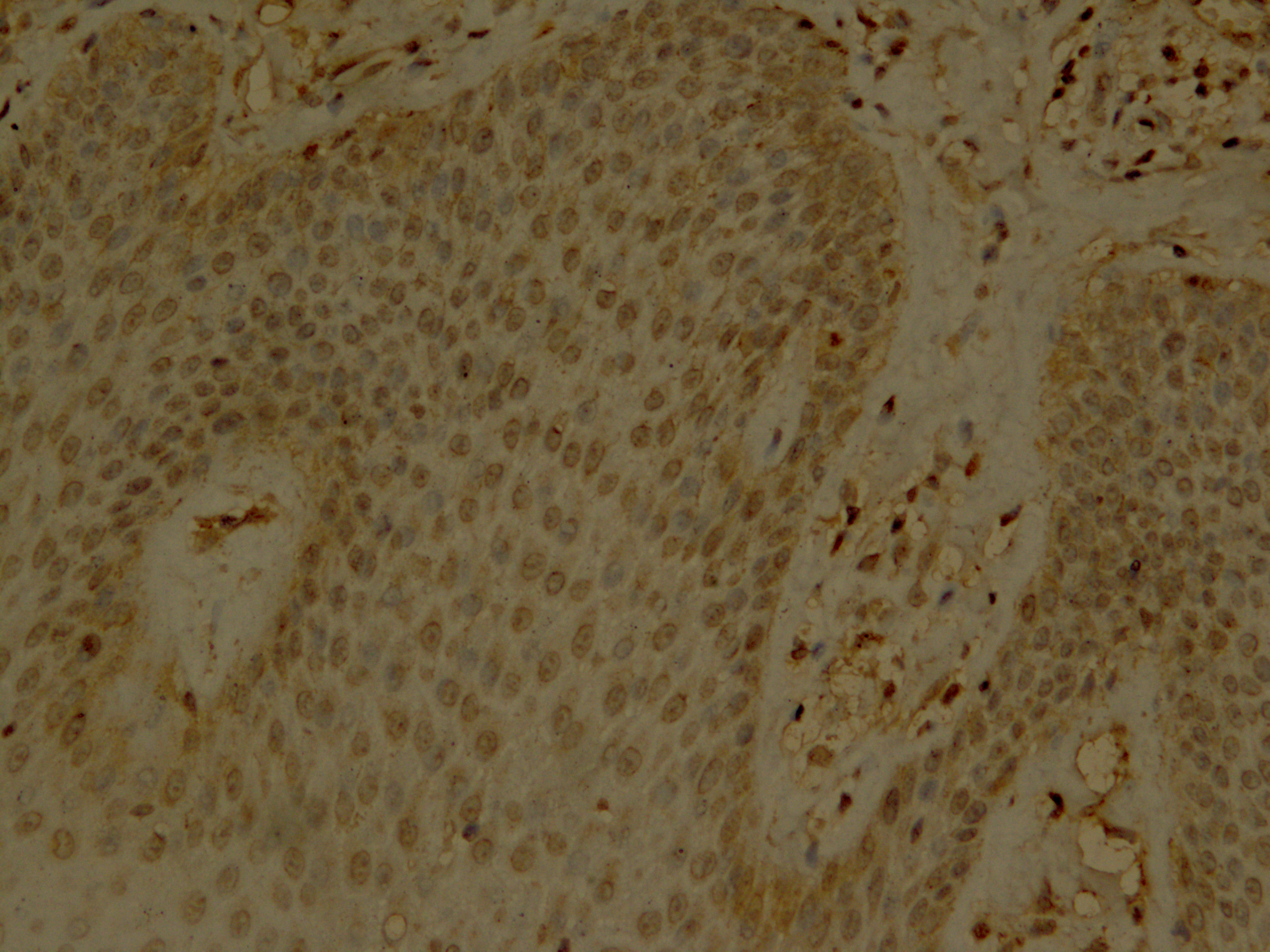

Supplement: Supplemental Information 10 [file peerj-12-16768-s010.tif]

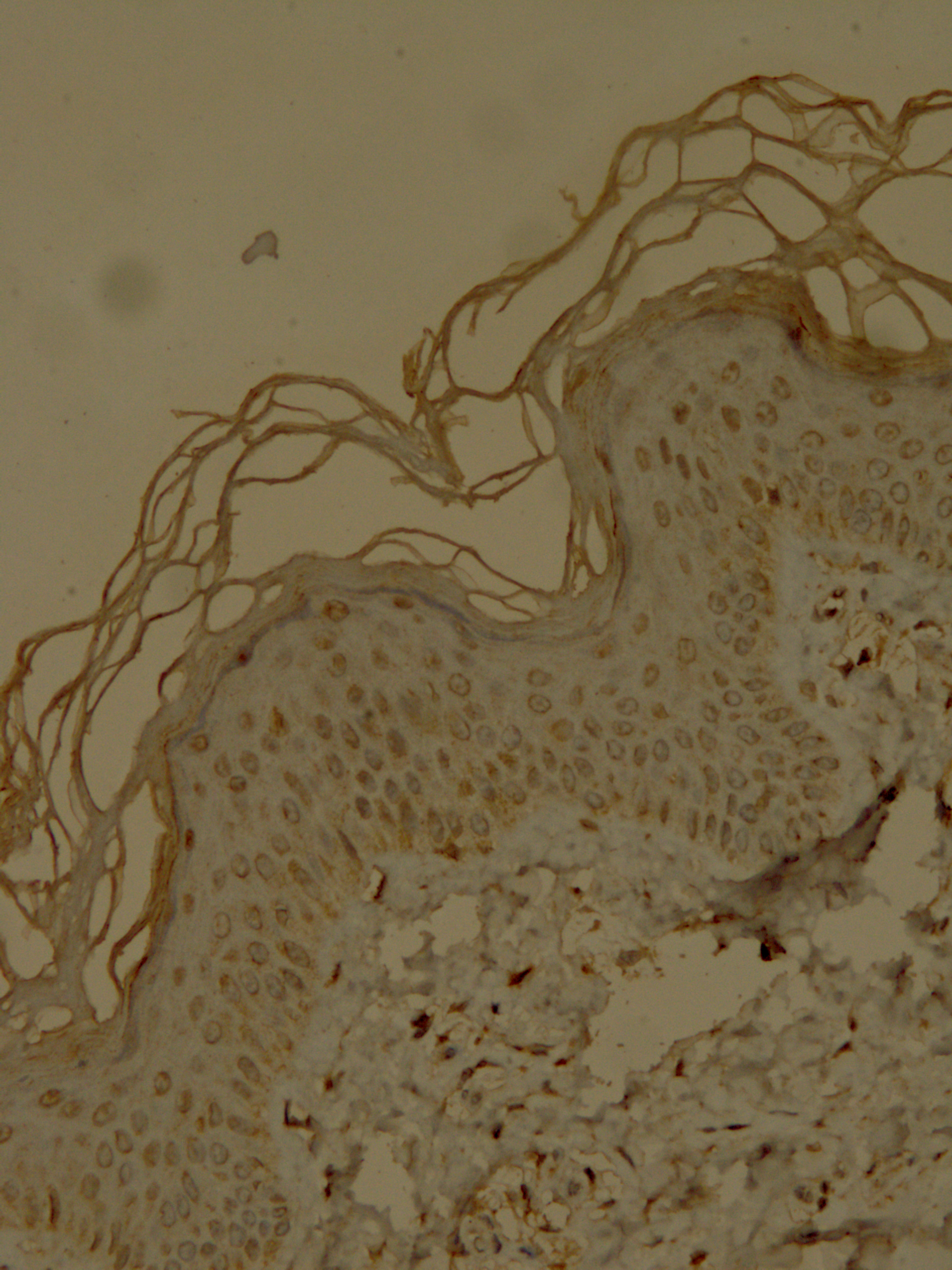

Supplement: Supplemental Information 11 [file peerj-12-16768-s011.tif]

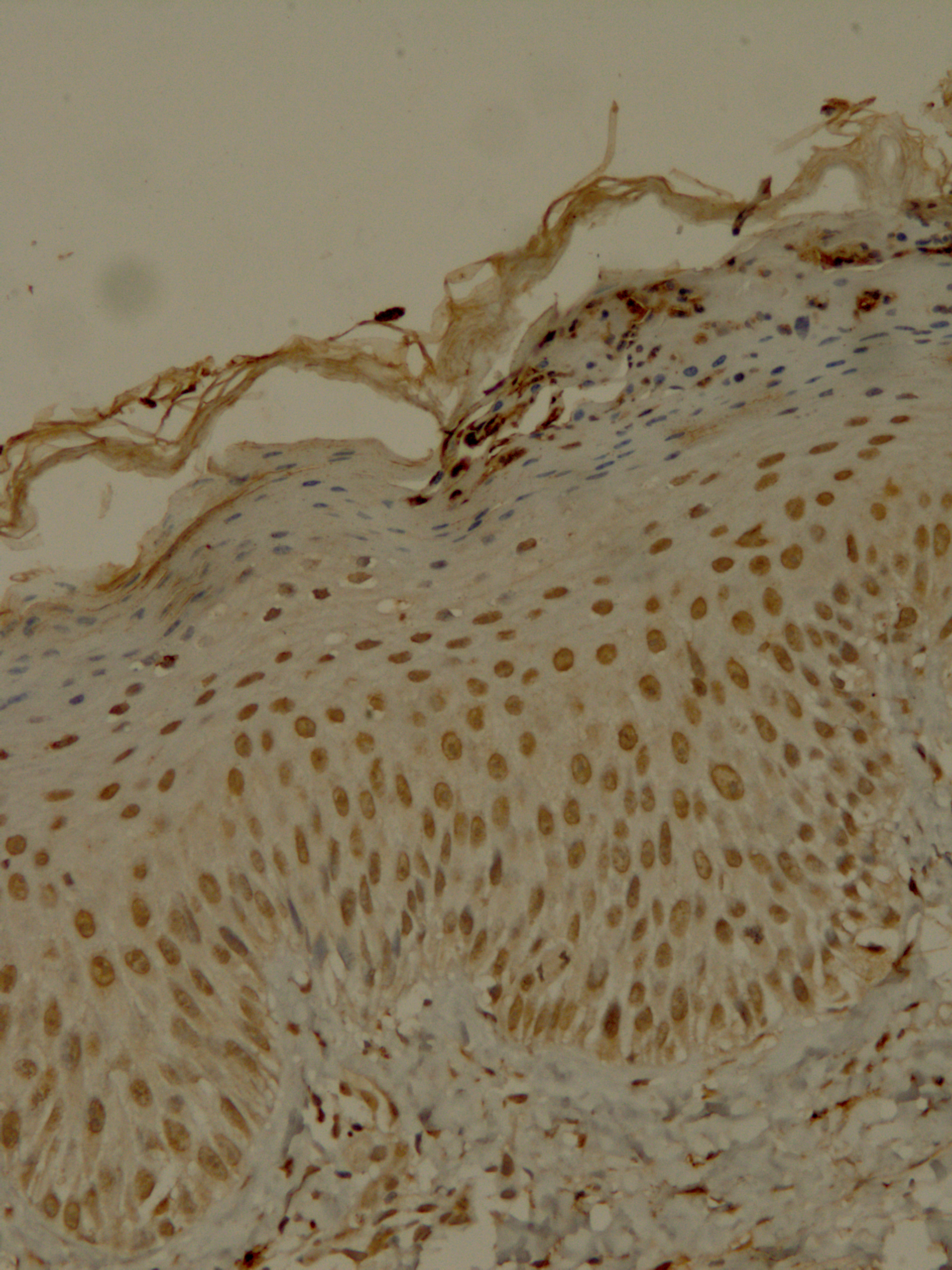

Supplement: Supplemental Information 12 [file peerj-12-16768-s012.tif]

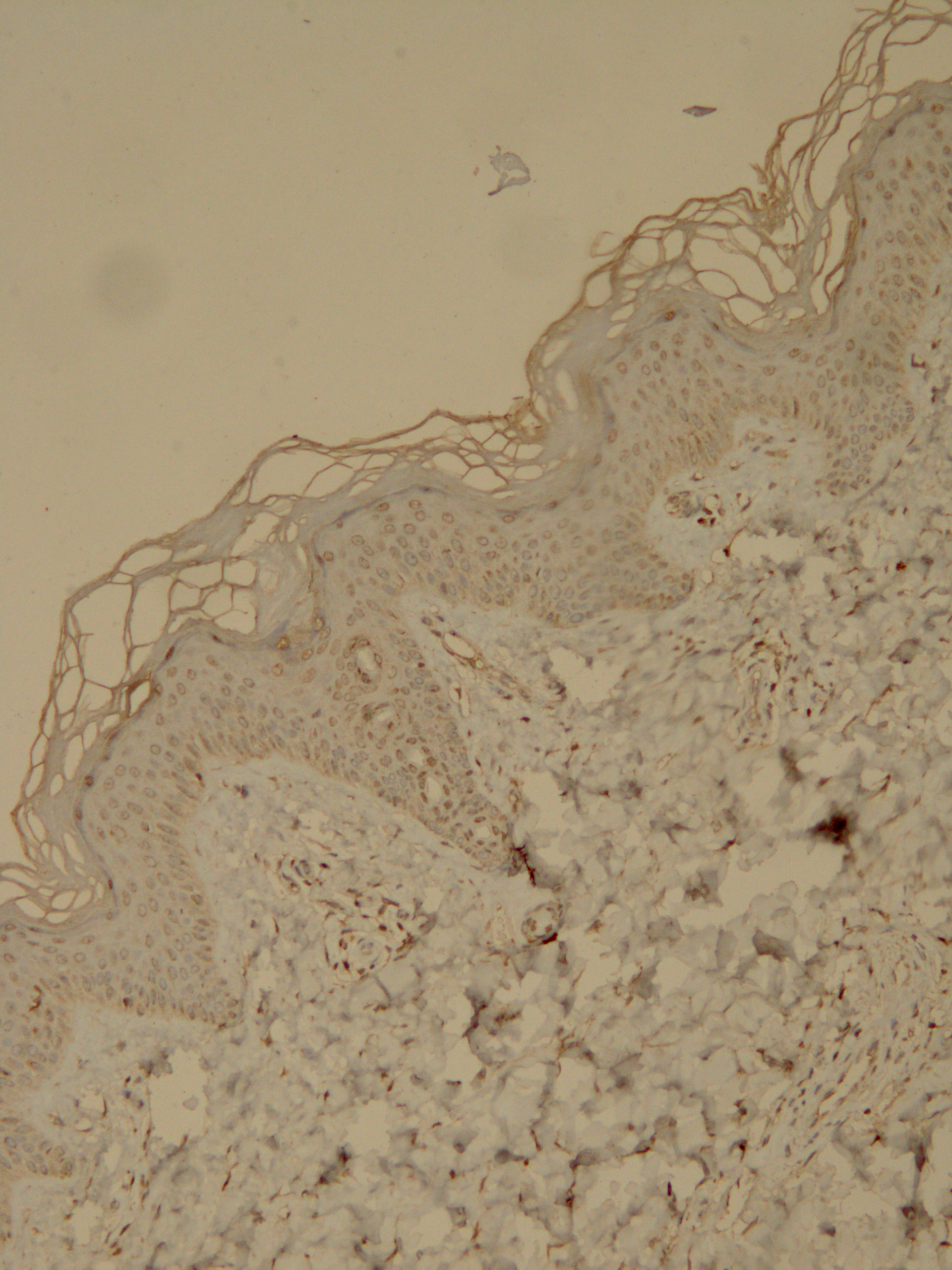

Supplement: Supplemental Information 13 [file peerj-12-16768-s013.tif]

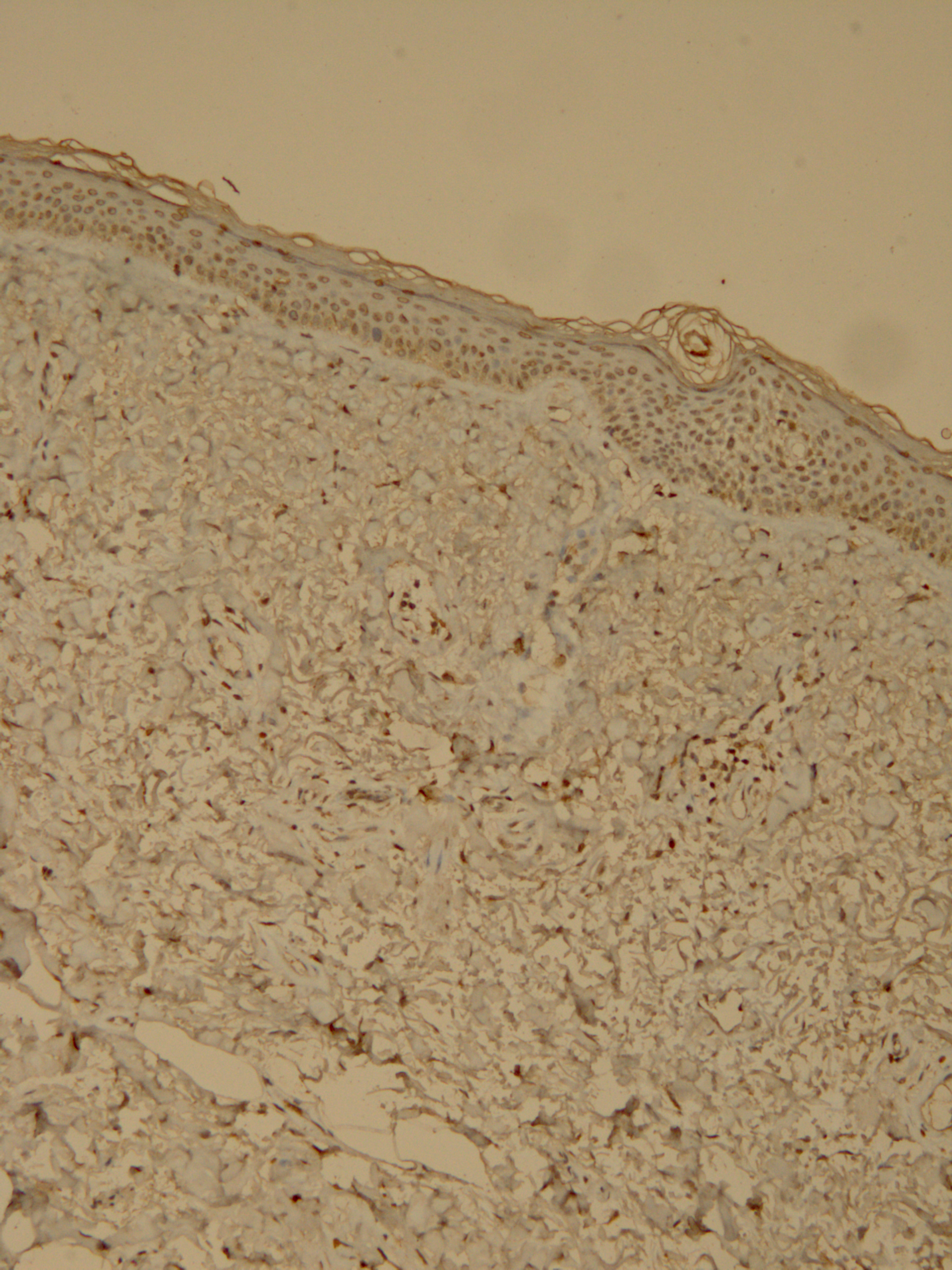

Supplement: Supplemental Information 14 [file peerj-12-16768-s014.tif]

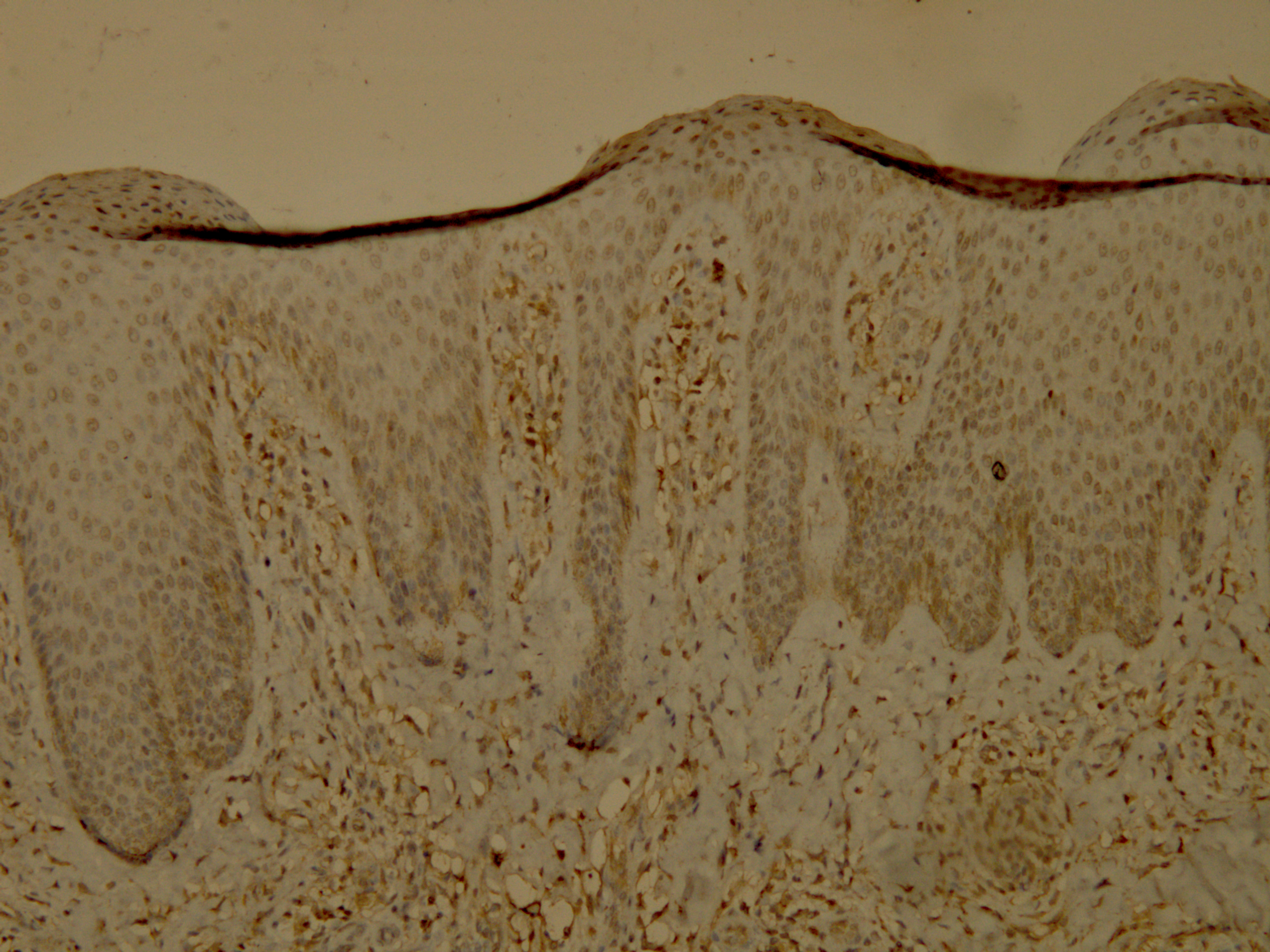

Supplement: Supplemental Information 15 [file peerj-12-16768-s015.tif]

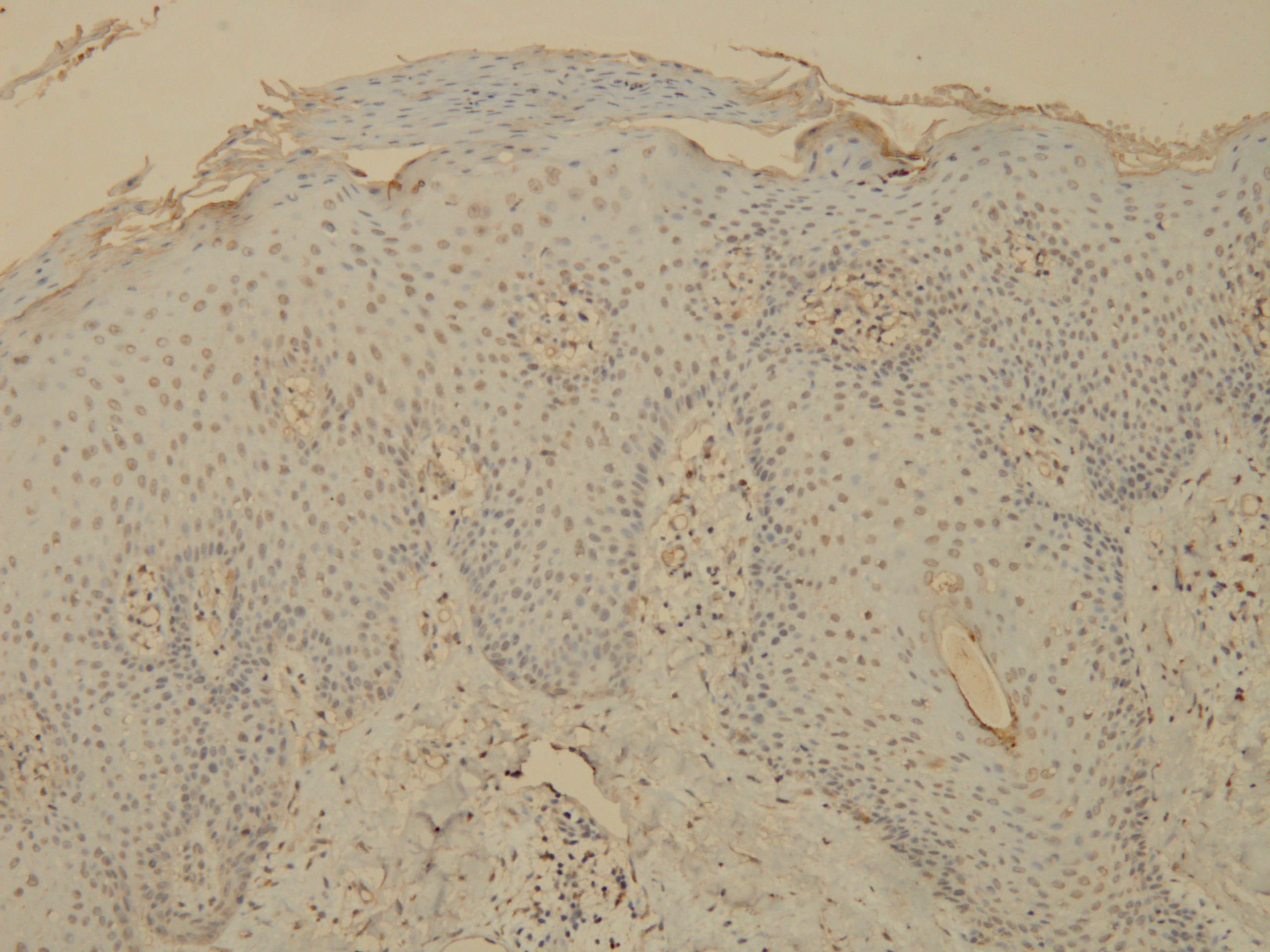

Supplement: Supplemental Information 16 [file peerj-12-16768-s016.tif]

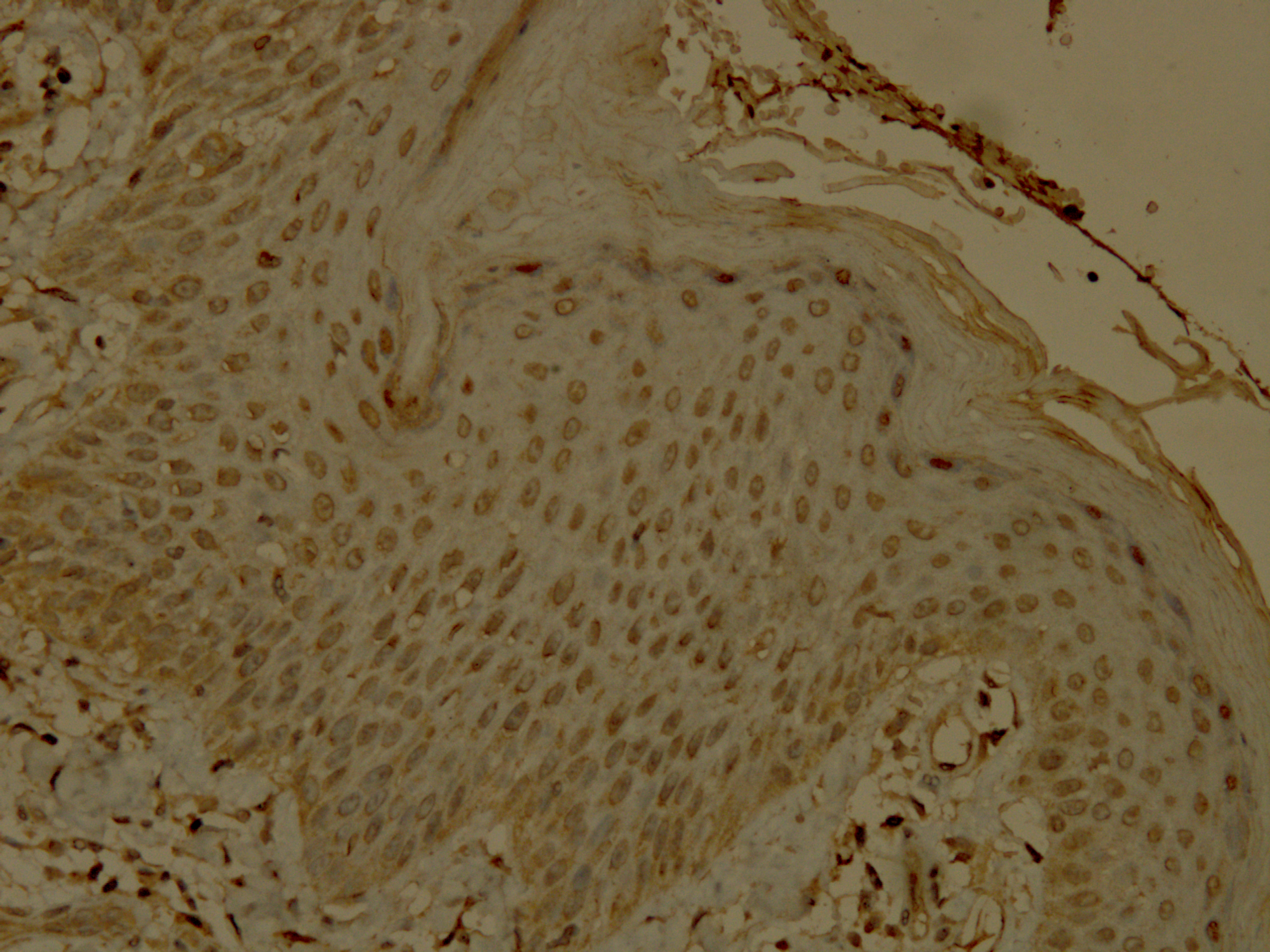

Supplement: Supplemental Information 17 [file peerj-12-16768-s017.tif]

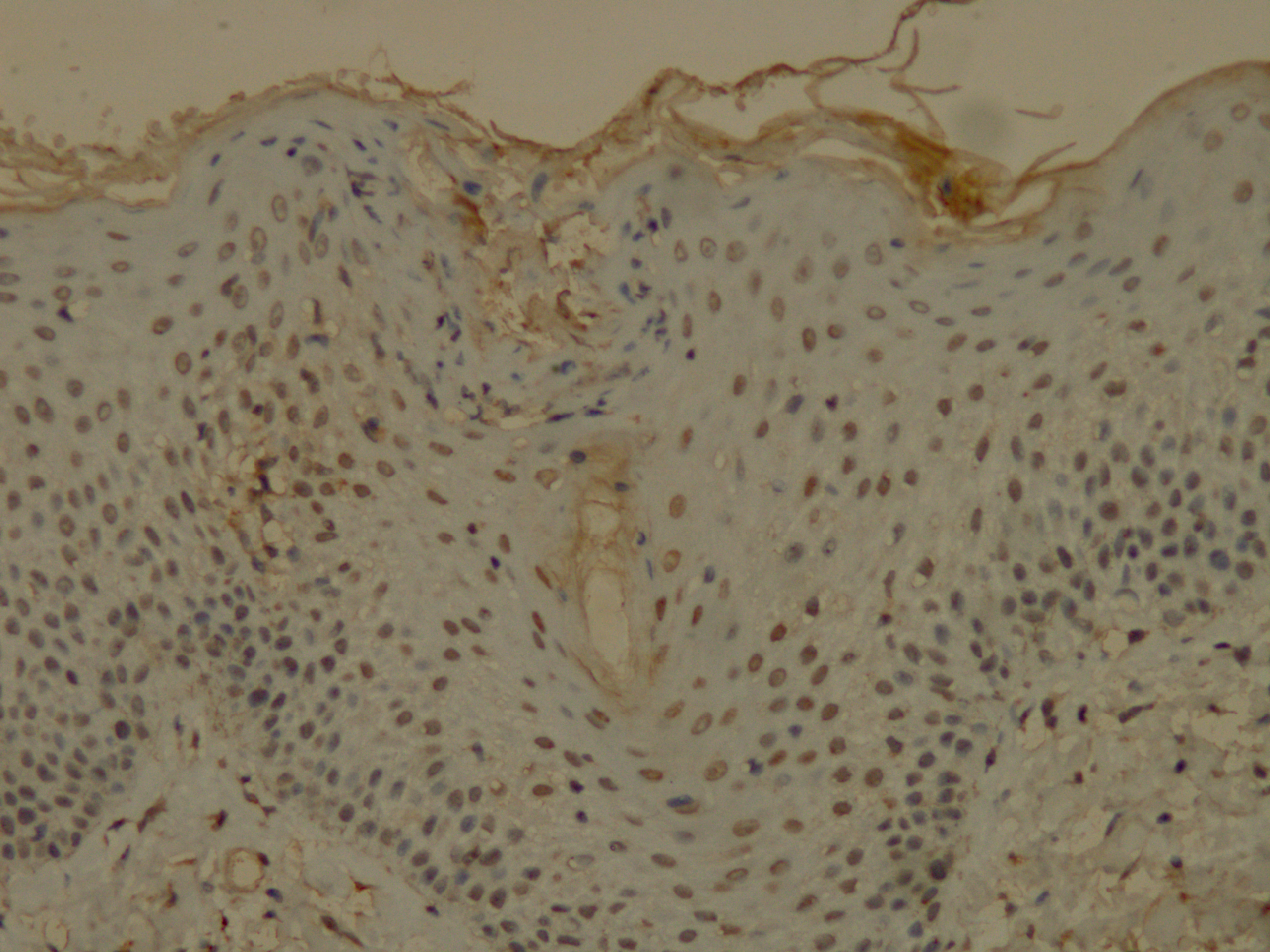

Supplement: Supplemental Information 18 [file peerj-12-16768-s018.tif]
